# Supplementary material for: Dynamics of a stochastic SEIQR model driven by Lévy jumps with bilinear incidence rates
Source: PLoS One. 2024 Jun 14;19(6):e0305139. doi: 10.1371/journal.pone.0305139 (PMC11178162; doi:10.1371/journal.pone.0305139)
Supplement: Attachment — Submitted filename: review_PONE-D-24-08895.pdf [file pone.0305139.s001.pdf]

Review on paper PONE-D-24-08895 submitted to PLOS
"Dynamics of a Stochastic SEIQR Model Driven by Lévy Jumps with Bilinear Incidence Rates"
by Qiuye Xia and Xiaoling Qiu

The paper studies a stochastic so called SEIQR infectious disease model driven by Lévy noise. SEIQR stands for the compartments S: Susceptible; E: Exposed; I: Infected; Q: Quarantined; R: Removed. The model equations (1) used by authors were introduced by Liu. The authors report the basic reproduction number R_0 of that system (formula below (1)). Here it would be highly useful for the reader if the authors could give a few more details how they obtained this result.

Then in Eq. (2) the authors add Lévy noise to the model equations. In the subsequent section the authors demonstrate uniqueness of the global positive solution.

I did not have the time to check the proofs, but the claimed features in Remark I appear to be sound. Finally the authors proof that for $R_0 > 1$ there is an asymptotically stable disease free equilibrium which is close to the one of the deterministic system (1). This appears quite reasonable. The presented results are quite interesting, especially Fig. 1 is showing the asymptotic solution fluctuating around the disease free state P_0. In Fig 2 the asymptotic solutions fluctuate around the endemic state of the deterministic model. Fig. 2 captures the situation where births and deaths are in equilibrium (or absent) whereas Fig. 1 corresponds to the situation when the mortality dominates. In a revised version the authors should explain this in more details and in simple words and add more physical interpretations, not only refer to theorems and proofs. In the "Conclusions " the authors should discuss the limitations of their model and some possible generalizations for future research. Overall the presented results appear to be sound and the inclusion of Lévy noise into a deterministic model is an interesting direction. Upon accounting for the enclosed comments, the paper can be recommended for publication in PLOS.

Further comments.
The English is quite sloppy. Expressions as "..won't explode.." (will not explode) or "..they're independent of each other.." (they are independent) should be improved. Apart of these remarks the reference list is rather poor. It is perfectly clear due to the immense literature of epidemic models that one can discuss but a few. However, the authors could at least discuss a bit more existing models related to their work, among them:

Compartment model with noisy transition rates: https://doi.org/10.1142/S0218127423500566

Non-markovian SIR model https://doi.org/10.1016/j.chaos.2022.112286

Stochastic SIS model https://doi.org/10.1016/j.physa.2019.121504

Stochastic multiple compartment model

https://journals.aps.org/pre/abstract/10.1103/PhysRevE.107.044207
