# Supplementary material for: Dynamics of a stochastic SEIQR model driven by Lévy jumps with bilinear incidence rates
Source: PLoS One. 2024 Jun 14;19(6):e0305139. doi: 10.1371/journal.pone.0305139 (PMC11178162; doi:10.1371/journal.pone.0305139)
Supplement: Attachment — Submitted filename: Report _2.pdf [file pone.0305139.s002.pdf]

Report on "Dynamics of a Stochastic SEIQR Model Driven by Lévy Jumps with Bilinear Incidence Rates"

This paper deals with the dynamics of a stochastic SEIQR model driven by Lévy jumps. It can be accepted for the publication after some major revisions.

Comments

1. The motivation of the choose of stochastic perturbations introduced in system (2) should be more justified.
2. Add the biological meanings of the integral terms used in system (2).
3. Study the persistence in the mean (see, Definition 4.1 in [1]).
4. Compare your model and results with others existing in the literature.
5. When infectious disease spreads into a population, individuals acquire knowledge about this disease. It will be interesting to investigate the memory effect on the dynamics of your model by using the new generalized fractional derivative and fractal-fractional derivative introduced in [2,3] instead of the classical time derivative used in (2).
6. Check the proof of Theorem 2.
7. Include and comment in the manuscript the recent and related works:
   [1] Qualitative analysis of a stochastic epidemic model with specific functional response and temporary immunity, Physica A 490 (2018).
   [2] A new mixed fractional derivative with applications in computational biology, Computation (2024).
   [3] A new class of generalized fractal and fractal-fractional derivatives with non-singular kernels, Fractal and Fractional (2023).
8. There are some typos. The authors should carefully read the manuscript.
9. Check and unify the citation of references.
