# Supplementary material for: Dynamics of a stochastic SEIQR model driven by Lévy jumps with bilinear incidence rates
Source: PLoS One. 2024 Jun 14;19(6):e0305139. doi: 10.1371/journal.pone.0305139 (PMC11178162; doi:10.1371/journal.pone.0305139)
Supplement: Attachment — Submitted filename: revision-response.pdf [file pone.0305139.s003.pdf]

Journal Name: PLOS ONE

Title: Dynamics of a Stochastic SEIQR Model Driven by Lévy Jumps with Bilinear Incidence Rates

Dear Editor,

We thank you very much for giving us an opportunity to revise our manuscript, we appreciate you and reviewers very much for your positive and constructive comments on our manuscript. We have studied reviewer's comments carefully and have revised the manuscript accordingly, and the detailed corrections are listed below point by point:

Answer to Reviewer 1

**Comments**	**Answers**
1.The motivation of the choose of stochastic perturbations introduced in system (2) should be more justified.	Thank you very much for your comment, and we have made additions according to your suggestion. The details are as follows(**see lines 58-63**): "However, disease can be affected by a variety of natural mutations, such as volcanic eruptions, chemical pollutants, and sudden climate changes, which are often not accurately described by stochastic models of Brownian motion. Therefore, many studies on natural mutation factors will use Lévy jump to describe. This perturbation can more accurately describe the impact of mutation factors, and more deeply understand and predict the trend of disease spread and development."
2. Add the biological meanings of the integral terms used in system (2).	Thank you for your valuable advice. The integral term here has no clear biological significance, but refers to the random disturbance suffered by the transmission of infectious diseases - the noise term. Noise can be understood as random, irregular physical or sound phenomena. A common noise model is a stochastic process, which describes the behavior of a set of random variables that change over time. By observing the noise amplitude distribution, the noise is transformed into a random process by using the concept of statistical correlation. However, the Levy process not only contains continuous changes, but also may include jumps, which can well describe some toxic pollutants, sudden climate changes, and vaccination phenomena, and is more universal than the Brownian movement. In the future, I will also consult relevant biological experts according to your suggestions to discuss the further correlation between stochastic process and biology
3. Study the persistence in the mean.	Thank you very much for your advice. I am in favor of adding the statement that the disease is persistent in the mean sense, and it has been supplemented in the revised version.(**see Theorem.7**) **Theorem 7** Assume that $S(t),E(t),I(t),Q(t),R(t)$ is the system (2) with initial value $(S(0),E(0),I(0),Q(0),R(0)) \in \Omega$ in solution, if $A > \frac{a+b}{\alpha(2a+b)}$, then $\lim\limits_{t\to\infty} \inf < I(t) >> 0$. In this case, the solution $I(t)$ of model (2) is durable in the sense of time mean.
4. Compare your model and results with others existing in the literature.	Thank you for your comments. We have added the innovation of this paper compared with previous papers in the introduction and conclusion.
5. When infectious disease spreads into a population, individuals acquire knowledge about this disease. It will be interesting to investigate the memory effect on the dynamics of your model by using the new generalized fractional derivative and fractal-fractional derivative introduced in [2,3] instead of the classical time derivative used in (2).	Thank you for your valuable advice. We have added references[2,3](**see line 236**). [2,3] applied fractional derivatives to computational biology, which is a important innovation in the subsequent infectious disease model research. They will provide great help for my subsequent research.

6. Check the proof of Theorem 2.	We have carefully calculated and checked Theorem 2 again, and have corrected the related errors in the proof of Theorem 2 in the modified version. Thanks for your suggestions.
7. Include and comment in the manuscript the recent and related works: [1] Qualitative analysis of a stochastic epidemic model with specific functional response and temporary immunity, Physica A 490 (2018). [2] A new mixed fractional derivative with applications in computational biology, Computation (2024). [3] A new class of generalized fractal and fractal-fractional derivatives with non-singular kernels, Fractal and Fractional (2023)	We appreciate your efforts to provide up-to-date articles related to the topic. According to your suggestion, and we have updated the corresponding references.
8. There are some typos. The authors should carefully read the manuscript.	Thank you for your advice. We double-checked the manuscript and carefully corrected the spelling mistakes.
9. Check and unify the citation of references.	Thank you for your correction. We unified the reference format and checked it carefully.

Answer to Reviewer 2

**Comments**	**Answers**
1. The abstract is poorly written, it need an improvement, for example, line 2 taking into account may be changed to considering and in line 4, Then, The must be change to Then.	Thank you very much for your review. Based on your comment, We have re-optimized the abstract section. The specific modifications are as follows:<br>"In this study, we propose a stochastic SEIQR infectious disease model driven by Lévy noise. Firstly, we study the existence and uniqueness of the global positive solution of the model by using the stop-time. Secondly, the asymptotic behavior of the stochastic system at disease-free equilibrium and endemic equilibrium. Then, the sufficient conditions for persistence under the time mean are discussed. Finally, our theoretical results are verified by numerical simulation."
2. The introduction should also make a compelling case for why the study is useful along with a clear statement of its novelty or originality by providing relevant information and providing answers to basic questions such as: i. What is already known in the literature? ii. What was done and how it was done?	Thank you very much for your review. We have added the purpose of introducing stochastic SEIQR model and revised the introduction to this article to give it a solid literature background and motivation according to your comment. The details are as follows:<br>" Tornatore et al.[4] proposed a stochastic SIR model with or without distributed time delay and studied the stability of disease-free equilibrium. Xu et al.[5] studied a kind of SIRS model, proved the existence and uniqueness of the positive solution of the model and obtained the conditions of disease extinction for epidemics. Zhao [6] studied the relationship between the threshold value of random SIRS model with saturation incidence and the extinction and persistence of epidemic diseases. Hieu [7] mainly studied the random SIRS model under telegraph noise and gave the conditions of disease persistence and disease-free equilibrium stability. Cai [8] mainly discussed the limit of transforming SDE model to discrete-time system and proved that the regeneration number can be used to judge the relevant properties of SDE model by using Markov semigroup theory. Yuguo et al. [9] analyzed that the distribution of random SIR Model solutions is absolutely continuous. Liu et al. [10] demonstrated that the system has a unique global positive solution and established sufficient conditions for disease persistence. Hattaf et al. [11] proposed and analyzed a stochastic SIR Epidemic model with specific functional response and time delay, and compared the difference of the basic regeneration number between the deterministic model and the stochastic model. Similarly, Lan et al. [12] studied a stochastic SIS model with saturated exposure rates and also found that the conditions for extinction of the disease were much weaker than the corresponding deterministic model. Ali and Khan [13,14] studied the dynamic properties of stochastic SEIR and SIRS models with saturation rate and simulated them using Legendre spectrum method."

3. In introduction section, provide theoretical justification for the choice of Lévy noise over other types of stochastic processes in modeling the abrupt fluctuations inherent in the disease transmission process?	Thank you very much for your suggestions. Based on your comment, we have explained the reasons for choosing Lévy noise in the introduction.The details are as follows(**see lines 58-63**):<br>"However, disease can be affected by a variety of natural mutations, such as volcanic eruptions, chemical pollutants, and sudden climate changes, which are often not accurately described by stochastic models of Brownian motion. Therefore, many studies on natural mutation factors will use Lévy jump to describe. This perturbation can more accurately describe the impact of mutation factors, and more deeply understand and predict the trend of disease spread and development."
4. In the section on the existence and uniqueness of the global positive solution provide more details on the mathematical intuition and biological implications behind the assumptions (H1) and (H2) related to the jump diffusion coefficient?	The jump section describes the sudden occurrence of random disturbances in the transmission of diseases - climate change, environmental pollution, etc. Among them, $C_i(Z)$ stands for pollution intensity, climate change amplitude, etc., which is essentially noise intensity The Lévy jump part described in (H1) is mainly used in proof. The existence and uniqueness theorem of the solution states that the Lipschitz condition needs to be satisfied, (H1) means that the jump part also satisfies the lipschitz condition. (H2) is used in the proof of Lemma 2, and is further optimized when the proof is checked again (H2).
5. How did you apply the Itô formula to derive the dynamics of your Lévy-driven stochastic differential equations? A step-by-step mathematical derivation would enhance the clarity. Add detailed mathematical derivations in the subsection discussing the application of the Itô formula.	Thank you very much for your advice. We have added Itô formula under Lévy process (**Lemma 1**), and supplemented the detailed calculation when applying Itô formula for the first time in this manuscript.The details are as follows:<br>**Lemma 1** (*Itô* formula) If $X(t)$ is the solution of a random differential equation<br>$$dx(t) = F(x(t),t)dt + G(x(t),t)dB(t) + \int_Z H(x(t),t,z)\tilde{N}(dt,dz).$$<br>If $V \in C^{2,1}(R^d \times [t_0,\infty];R_+)$, thus the random derivative of $V(x,t)$ is: $dV(x,t) = LV(x,t)dt + V_x(x,t)G(x(t),t)dB(t) + \int_Z [V(x+H(x,t,z)) - V(x,t)]\tilde{N}(dt,dz)$, where<br>$$\begin{aligned} LV(x,t) =& V_t(x,t) + V_x(x,t)F(x,t) + \frac{1}{2}trace[G^T(x,t)V_{xx}G(x,t)] \\ &+ \int_Z [V(x+H(x,t,z)) - V(x,t) - V_x(x,t)H(x,t,z)]v(dz). \end{aligned}$$
6. How was the specific form of the Lyapunov function chosen, and what are the mathematical criteria for its selection in proving the asymptotic behavior around the disease-free and endemic equilibrium?	Thank you for your review. In the process of writing the manuscript, it was very difficult to find the Lyapunov function. By reading a lot of literature, we analyze the previous low-dimensional models and derive the Lyapunov function required for this model. Further verify whether the corresponding Lyapunov function is reasonable and effective when applied to the model, and finally determine the appropriate Lyapunov function.
7. Again, in section on the existence and uniqueness of the global positive solution, what mathematical techniques were employed to ensure the solution stays positive and within the biologically feasible region, given the presence of jumps and discontinuities.	First, by setting the condition (H1), we ensure that the jump part also satisfies the local Lipsitz condition, that is, the condition required by the existence and uniqueness theorem of the local solution. For general nonlinear systems, the global solution can only exist in a small range of time t, which means that the system equilibrium solution is not globally attractive, it will lose normality in finite time and produce singularity, that is, the solution itself or some derivatives tend to infinity with the increase of time. Therefore, we use the blasting time proof to understand how to go from local to global. When an asymmetric system has a solution, its blasting time tends to infinity, and the system solution is unique for all time $t \geq 0$.
8. The conditions for stochastic stability mentioned are quite specific. Provide more insight into how these conditions ensure the stability of the disease-free and endemic equilibrium points? This should be addressed in both the sections on the disease-free equilibrium and the endemic equilibrium analysis.	We agree that the source of the stochastic stability condition should be explained in the course of the proof(**see lines 150,181**). Thank you again for your valuable comments. I will ensure that these recommendations are incorporated and that the conclusions are improved accordingly.

9. What numerical methods were used for the simulations shown in Figures 1 and 2, and how do these methods accurately capture the effects of Lvy jumps?	Thank you for your review. Both Figure 1 and Figure 2 invoke Lévy functions in the main model, and the main model mainly adopts difference iteration to simulate random differential equations.
10. The references are very few, the author may add some recent related work. They may also add the following recent work, but not mandatory. https://doi.org/10.3934/math.2023210 https://doi.org/10.1080/07362994.2021.1981382 https://doi.org/10.3390/sym14091838 https://doi.org/10.1007/s11071-021-07095-7.	We appreciate your efforts to provide up-to-date articles related to the topic. According to your suggestion, and we have updated the corresponding reference. They have been shown in **references[13], [14], [20]**.
11. Author may look for some punctuation, typos and editing issues.	Thank you very much for your careful review. We are very sorry for our incorrect writing. We have checked the manuscript carefully and corrected the punctuation, typos and editing issues.

Answer to Reviewer 3

**Comments**	**Answers**
1. The authors report the basic reproduction number R_0 of that system (formula below (1)). Here it would be highly useful for the reader if the authors could give a few more details how they obtained this result.	Thank you for your valuable advice. We have detailed the basic reproduction number calculation in the modified version. The details are as follows:<br>Converting model (1) to the following form:<br>$\frac{dx_i}{dt} = f_i(x) = r_i(x) - h_i(x) = \begin{pmatrix} \alpha SI \\ 0 \\ 0 \\ 0 \\ 0 \end{pmatrix} - \begin{pmatrix} (a+b)E \\ (d+h+\delta)I - bE \\ kQ - hI \\ \alpha SI + \mu S - A \\ nR - \mu S - aE - dI - kQ \end{pmatrix}$ $, i = 1,2...m$ Let $F = \left[\frac{\partial r_i}{\partial x_j}\right]$, $V = \left[\frac{\partial h_i}{\partial x_j}\right]$, where $1 \leq i,j \leq m$, FV^{-1} is called a regenerative matrix. The basic regeneration number is the spectral radius of the regeneration matrix. Basic reproduction number R_0 of system (1) is<br>$$\frac{bk\alpha A}{\mu(a+b)(d+h+\delta)}.$$
2. In Fig 2 the asymptotic solutions fluctuate around the endemic state of the deterministic model. Fig. 2 captures the situation where births and deaths are in equilibrium (or absent) whereas Fig. 1 corresponds to the situation when the mortality dominates. In a revised version the authors should explain this in more details and in simple words and add more physical interpretations, not only refer to theorems and proofs.	Thank you very much for your advice. We have supplemented the physical meaning of Fig 1 and Fig 2 in the revised version(**see lines 164-167,194-196**). The details are as follows:<br>"From the observation of Fig 1, it can be seen that under certain conditions of parameters, the system will stabilize in a situation where only susceptible persons exist. The infected person, exposed person of the virus will disappear, which means the disease will disappear."<br>"It can be seen from Fig.2 that, under certain parameter conditions, although the proportion of recovered patients is obvious, the disease will continue because exposed persons and infected persons will still exist in a certain proportion."

3. In the Conclusions the authors should discuss the limitations of their model and some possible generalizations for future research.	We agree that the conclusion part of the paper should be expanded, and in the revised conclusion part, the future research direction is added. Thank you again for your valuable comments. I will ensure that these recommendations are incorporated and that the conclusions are improved accordingly(**see lines 231-239**). The details are as follows: "Different from other three-compartment and four-compartment models, this paper proposes to add isolation compartment and introduce Lévy noise random interference, respectively proving the stability of the equilibrium point and the conditions for the continuous existence of the disease, providing a theoretical basis for the subsequent control of infectious diseases. However, when an infectious disease spreads through a population, the individual gains knowledge about the disease. The classical time derivative cannot reflect the memory effect of model dynamics. The time derivative in this paper is replaced by a fractional derivative [21,22], and delayed feedback [23] is considered for factors such as vaccines in random infectious diseases. At the same time, we can consider the general non-Markov SEIQR model and compare the discrete and continuous time versions.[24] "
4. The English is quite sloppy. Expressions as ..wont explode.. (will not explode) or ..theyre independent of each other.. (they are independent) should be improved.	Thank you very much for your careful review. We are very sorry for our incorrect writing. We have checked the manuscript carefully and corrected the typos .
5. Apart of these remarks the reference list is rather poor. It is perfectly clear due to the immense literature of epidemic models that one can discuss but a few. However, the authors could at least discuss a bit more existing models related to their work, among them: (1) Compartment model with noisy transition rates: https://doi.org/10.1142/S0218127423500566 (2) Non-markovian SIR model: https://doi.org/10.1016/j.chaos.2022.112286 (3) Stochastic SIS model: https://doi.org/10.1016/j.physa.2019.121504 (4) Stochastic multiple compartment model: https://journals.aps.org/pre/abstract/10.1103/PhysRevE.107.044207	We appreciate your dedication to providing up-to-date articles related to this topic. According to your suggestion, we have updated the corresponding reference material.(**see references [12], [23], [24]**)

At this time, one can see that the quality of this paper has been improved. We express our great thanks again to you and the reviewer. The revised manuscript and revision-response have been uploaded to your journal. We hope that the corrections and revisions will be satisfactory and that the revised version will be accepted for publication in your journal.

Best regards,

Yours sincerely,

Xiaoling Qiu
